# Inhibitory Effect of* Cuphea aequipetala* Extracts on Murine B16F10 Melanoma* In Vitro* and* In Vivo*

**DOI:** 10.1155/2019/8560527

**Published:** 2019-05-29

**Authors:** Ashanti Concepcion Uscanga-Palomeque, Pablo Zapata-Benavides, Santiago Saavedra-Alonso, Diana Elisa Zamora-Ávila, Moisés Armides Franco-Molina, Mariela Arellano-Rodríguez, Edgar Manilla-Muñoz, Ana Carolina Martínez-Torres, Laura M. Trejo-Ávila, Cristina Rodríguez-Padilla

**Affiliations:** ^1^Universidad Autónoma de Nuevo León, Facultad de Ciencias Biológicas, Laboratorio de Inmunología y Virología, 66455 San Nicolas de los Garza, NL, Mexico; ^2^Universidad Autónoma de Nuevo León, Facultad de Medicina Veterinaria y Zootecnia, Departamento de Virología, 66050 Cd Gral Escobedo, NL, Mexico

## Abstract

*Cuphea aequipetala* (*C. aequipetala*) has been used in Mexican traditional medicine since prehispanic times to treat tumors. In this paper, we evaluated the antiproliferative and apoptotic effect of the methanolic and aqueous extracts of* C. aequipetala* on several cancer cell lines including the B16F10 cell line of murine melanoma and carried a murine model assay.* In vitro* assay analyzed the effect in the cellular cycle and several indicators of apoptosis, such as the caspase-3 activity, DNA fragmentation, phosphatidylserine exposure (Annexin-V), and induction of cell membrane permeabilization (propidium iodide) in the B16F10 cells.* In vivo*, groups of C57BL/6 female mice were subcutaneously injected with 5x10^5^ B16F10 cells and treated with 25 mg/mL of* C. aequipetala* extracts via oral. Aqueous and methanolic extracts showed a cytotoxic effect in MCF-7, HepG2, and B16F10 cell lines. The methanolic extract showed more antiproliferative effect with less concentration, and for this reason, the* in vitro* experiments were only continued with it. This extract was able to induce accumulation of cells on G1 phase of the cell cycle; moreover, it was able to induce DNA fragmentation and increase the activity of caspase-3 in B16F10 cells. On the other hand, in the murine model of melanoma, the aqueous extract showed a greater reduction of tumor size in comparison with the methanolic extract, showing an 80% reduction versus one of around 31%, both compared with the untreated control, indicating a better antitumor effect of* C. aequipetala* aqueous extract via oral administration. In conclusion, the* in vitro *data showed that both* C. aequipetala* extracts were able to induce cytotoxicity through the apoptosis pathway in B16F10 cells, and* in vivo,* the oral administration of aqueous extract reduces the melanoma tumoral mass, suggesting an important antitumoral effect and the perspective to search for effector molecules involved in it.

## 1. Introduction

Malignant melanoma is the most aggressive form of skin cancer [1, 2], because of its ability to metastasize to other organs such as lungs, liver, brain, or lymph nodes [3, 4]. The global incidence of melanoma has been steadily increasing in the last decades [5, 6], affecting mainly white men [7] with an average age at diagnosis of 57 years and a 5-year survival of around 5% to 19% in late stages of diagnosis [8]. Morbidity and mortality are primarily associated with metastatic disease and poor responses to most standard chemotherapies [8]. Due to these problems, efforts to find new and better alternatives to increase the survival of patients with melanoma are a necessity.

From this perspective, plant-derived compounds have received considerable attention in recent years because of their pharmacological properties, such as cytotoxicity and chemotherapeutic activities in cancer [9, 10]. Indeed, the main drugs currently used for cancer treatment have been isolated from natural sources, which include taxol from Taxus species, vinblastine and vincristine from* Catharanthus roseus*, topotecan and irinotecan from* Camptotheca acuminata*, and etoposide and teniposide from* Podophyllum peltatum* [11].

For that reason, several studies using herbal products have been carried out against cancer cells, displaying different effects, for example, products from Forsythiae fructus inhibit cell proliferation by involving MAPKs/Nrf2/HO-1 pathway [12]. Ganoderma lucidum methanolic extract has antioxidant and anti-inflammatory activity, induces caspase-dependent apoptosis, cell death mediated by upregulated p53, and inhibits Bcl-2 expression [13]. Extracts of Taohong siwu inhibit angiogenesis by downregulating of VEGF [14, 15], and Spatholobus suberectus extracts inhibit of the process of adhesion, invasion, migration, and metastasis of cancer [16].

Therefore, we decided to work with* Cuphea aequipetala* (Lythraceae), a perennial plant native to Mexico that grows in the open and humid fields of pine-oak woods at 2000-2540 m above sea level [17]. In Mexico,* C. aequipetala* is also known as “cancer weed” and has been used since prehispanic times for the treatment of different diseases. The aqueous extracts of the aerial parts of the plant showed strong activity against stomach ailments such as pain, burning sensation, and infections by* Helicobacter pylori* [18, 19], as well as showing cytotoxic activity against different types of cancer cell lines, such as HEp-2 (human larynx carcinoma), HCT-15 (human colon cancer), and DU-145 (human prostate carcinoma) [20]. In this work we evaluated the cytotoxic effect of* C. aequipetala* in several cancer cell lines, and then carried an* in vivo* model to analyze the antitumor effects of the methanolic and aqueous extracts of this plant against murine melanoma B16F10 cells.

## 2. Materials and Methods

### 2.1. Plant Material

Mature and fresh* Cuphea aequipetala* plant was obtained from the Sonora market in Mexico City; the sample was then identified and authenticated by the botany department of the Facultad de Ciencias Biológicas, Universidad Autónoma Nuevo León (UANL), with accession number 27909, and was placed at the herbarium.

### 2.2. Cuphea aequipetala Aqueous Extract

Dried, ground, aerial parts (10 g) were selected, and the chemical substances were extracted with 100 mL of distilled water at 80°C for 15 min, the insoluble material was removed by centrifugation at 5,000 rpm for 10 min, filtered through a Whatman no.1 filter paper, and collected in a glass beaker. The aqueous extract was freeze-dried and a powder was obtained. The extract was stored in an airtight container in the cold room at 4°C until further testing, and dried crude extracts were dissolved in distilled water and filtered out for sterilization obtaining a stock solution of 25 mg/mL.

### 2.3. *Cuphea aequipetala* Methanolic Extract

The dried and ground aerial parts of the plant (10 g) were selected, and the chemical substances were extracted by maceration for 48h with 100 mL of 96% methanol at room temperature. The extract was filtered using a Whatman no.1 filter paper, and the collected methanol extract was evaporated by incubation at 50°C to produce a red-brown residue. The extract was stored in an airtight container in the freezer at -80°C until further testing, and dried crude extracts were dissolved in distilled water and filtered out for sterilization obtaining a stock solution of 25 mg/mL.

### 2.4. Cell Line and Culture

B16F10, MCF-7, and HepG2 cancer cells lines were purchased from American Type Culture Collection (ATCC) (Manassas VA, USA). The cells were maintained in Dulbecco's Modified Eagle Medium: Nutrient Mixture F-12 (DMEM/F12) (Invitrogen, USA), supplemented with 10% fetal bovine serum (FBS) (Invitrogen, USA), 100 IU/mL penicillin, and 100 *μ*g/mL streptomycin in a humidified incubator containing 5% CO_2_ at 37°C.

### 2.5. Cell Viability Assay

Cell viability was evaluated using MTT [3-(4,5-dimethylthiazol-2-yl)-2,5-diphenyltetrazolium bromide] assay. We seeded 5×10^3^ cells in 100 *μ*L of culture medium into 96-well plates in triplicate and cultured overnight. Then, the medium was replaced with fresh medium containing different concentrations of the aqueous (0.05, 0.1, 0.2, 0.4, 0.6, and 0.8 mg/mL) and methanolic extracts (0.0425, 0.085, 0.17, 0.34, 0.51, and 0.68 mg/mL) and incubated for 48h. After incubation, 20 *μ*L of MTT solution (0.5 mg/mL in phosphate-buffered saline) was added to each well followed by 1h of incubation. The medium was discarded, and 100 *μ*L of dimethyl sulfoxide was added to each well and incubated for 20 min. The optical density (OD) was determined at 570 nm using a microplate reader (Microplate Autoreader EL311, BioTek Instruments Inc., Winooski, VA, USA). Each experiment was done in triplicate and results are expressed as mean ± SD.

### 2.6. Viability of Peripheral Blood Mononuclear Cells (PBMC)

To determine the cytotoxicity of the extracts in normal human cells, a peripheral EDTA blood sample was collected from healthy donors and mononuclear cells were isolated by gradient using Ficoll-Histopaque-1119 (Sigma-Aldrich, St Louis, MO, USA) and centrifugated at 1600 rpm for 30 min at room temperature. Then, the cells were washed twice with PBS (phosphate-buffered saline) at 1600 rpm for 10 min and the supernatant was discarded. Immediately, the pellet was resuspended in RPMI 1640 medium supplemented with 10% FBS and antibiotic-antimycotic (Invitrogen USA). All samples were carried out by triplicate in 96-well plates, adjusted to 2.5 x 10^6^ cells per well, treated with several concentrations of aqueous (0.05, 0.1, 0.2, 0.4, 0.6, 0.8, and 1.6 mg/mL) and methanolic extracts (0,042, 0.085, 0.17, 0.34, 0.51, 0.68, and 1.36 mg/mL), and incubated for 24 h in a humidified incubator containing 5% CO_2_ at 37°C. After incubation, 20 *μ*L of MTT solution (0.5 mg/mL in PBS) was added to each well followed by 1h of incubation. The plate was centrifuged at 1600 rpm for 10 minutes at room temperature and the medium was discarded; 100 *μ*L of dimethyl sulfoxide was added to each well and incubated for 20 min. The optical density (OD) was determined at 570 nm using a microplate reader (Microplate Autoreader EL311, BioTek Instruments Inc., Winooski, VA, USA). Each experiment was done in triplicate and results are expressed as mean ± SD.

### 2.7. Colony Forming Units Assay

B16F10 cells were seeded in 6-well plates, 200 cells to the untreated control, and 500 cells for every concentration of the methanolic extract. After 24h, the methanolic extract was added in different concentrations; 24h after incubation the treatment was removed, and the cells were supplemented with fresh medium without treatment. After 8 days, the cells were washed with PBS and the colonies were fixed with 100% ethanol and then stained with 1 mL/well of crystal violet solution (0.1% in PBS) for 15-20 min. To determine the number of colony forming units (CFU), the clones were observed and counted in the stereoscopic microscope. Formulas used to determine the CFU were as described by Franken et al., 2005. Plated Efficiency = (Number of colonies counted in the control/ Number of cells plated) x 100 and Surviving Fraction = (Number of colonies counted after treatment/ Number of cells plated x PE). Each different concentration was carried out in triplicate.

### 2.8. Cell Cycle Analysis

To evaluate the cell cycle, 1x10^6^ B16F10 cells were seeded in 6-well dishes, exposed to the CC_50_ dose of the methanolic extract (0.2 mg/mL) or etoposide as the positive control, and incubated for 24, 48, and 72h. The cells were collected and washed with PBS; the pellets were then resuspended in 5 mL of cold 70% ethanol and fixed overnight at 4°C. The fixed cells were washed twice in PBS and then incubated with 50 *μ*L of RNase solution (10 *μ*g/mL in sterile PBS) and 25 *μ*L of propidium iodide (1 mg/mL in sterile PBS) for 30 min at room temperature in the dark. Flow cytometric analysis was performed by a BD AccuriC6 flow cytometer (BD Biosciences, Franklin Lakes, NJ, USA). For each analysis, 10,000 events were collected and analyzed using FlowJo software (LLC, Ashland, Oregon USA).

### 2.9. Cell Death Analysis

The B16F10 cells were cultured at a density of 4x10^4^ in a 24-well plate. After 24 h, the cells were exposed to one concentration of methanolic extract (0, 0.135 mg/mL) and incubated at different times (24 and 48 h). Etoposide (Enzo Life Sciences, NY, USA) at 1.5 mM was used as a control of apoptosis induction. After treatment, the cells were harvested and washed twice with cold PBS and suspended in 100 *μ*L of binding buffer (0.1 M Hepes/NaOH pH 7.4, 1.4 M NaCl, and 25 mM CaCl_2_) supplemented with 0.5 *μ*L of APC-Annexin-V and 0.5 *μ*L of propidium iodide (PI). The cell suspension was gently vortexed and incubated for 20 min at 4°C in the dark to be assessed by flow cytometry in a BD AccuriC6 flow cytometer (BD Biosciences, Franklin Lakes, NJ, USA) (total population 10,000 cells). Data was analyzed using FlowJo software (LLC, Ashland, Oregon, USA).

### 2.10. DNA Fragmentation Assay

B16F10 cells (1x10^6^) were seeded in 6-well dishes and treated with 0.2 mg/mL of* C. aequipetala* extract as described above, and 72h after treatment, the cells were harvested for DNA fragmentation analysis using the Quick Apoptotic DNA Ladder Detection kit (BioVision Research, Mountain View, California, USA) as per the manufacturer's protocol. DNA extraction was analyzed by 1% agarose gel electrophoresis and stained with ethidium bromide and visualized using an ultraviolet transilluminator (Spectroline Bi-O-Vision, San Diego, CA, USA).

### 2.11. Analysis of Caspase-3 Activity

B16F10 cells (1X10^6^) were treated with 0.2 mg/mL of* C. aequipetala* methanolic extract, and 72h after, cells were analyzed for caspase-3 activity using a colorimetric activity assay kit (Chemicon International Inc., Temecula, CA) according to manufacturer's instructions and as described by Zamora-Avila and collaborators. In brief, cells were resuspended in 200 *μ*L of 1X cell lysis buffer, incubated on ice for 10 min, and centrifuged at 10,000 g. The assays were prepared in 96-well plates (5X assay buffer, caspase-3 sample, deionized water, and caspase-3 substrate) and included a blank substrate (5X assay buffer, deionized water, and caspase-3 substrate). Samples were incubated for 2h at 37°C and finally read at 405 nm in a microplate reader (Microplate Autoreader EL311, BioTek Instruments Inc. Woonsocket, RI USA) [21]. The caspase-3 activity was determined by comparing the OD differences between treated and untreated cells. Each experiment was done in triplicate and results are expressed as mean ± SD.

### 2.12. *In Vivo* Model Assay

In this study, the experimental protocols were approved by the Institutional Animal Care and Use Committee of the College of Biological Sciences, UANL. Eight-week-old C57BL/6 female mice were purchased from Harlan Laboratory (Coyoacán, CDMX, México). Mice were caged under controlled room temperature, humidity, and light (12/12h light-dark cycle), with water and food ad libitum. Eighteen mice were given subcutaneous injections of 5x10^5^ B16F10 cells into the posterior left flank. Three days later, mice were separated into three groups with six mouse each: (i) untreated group (regular water); (ii) methanolic group (treated with 25 mg/mL of* C. aequipetala* methanolic extract), and (iii) aqueous group (treated with 25 mg/mL of* C. aequipetala* aqueous extract); all the treatments were given as drinking water, changing it daily for 14 days. Body weight and survival of mice were monitored daily.

### 2.13. Statistical Analysis

Independent experiments were repeated three independent times. Mice were randomly assigned to different groups for* in vivo* study. Mann-Whitney and two-tailed unpaired Student's* t*-tests were performed using GraphPad Prism Software (San Diego CA, USA) and presented as mean values ±SD. P-values were considered significant as follows:* p *< 0.05 (*∗*),* p* < 0.01 (*∗∗*), and* p* < 0.001 (*∗∗∗*).

## 3. Results

### 3.1. Effect of Aqueous and Methanolic Extracts of* Cuphea aequipetala* in Cancer Cells Lines

**Figure 1:**
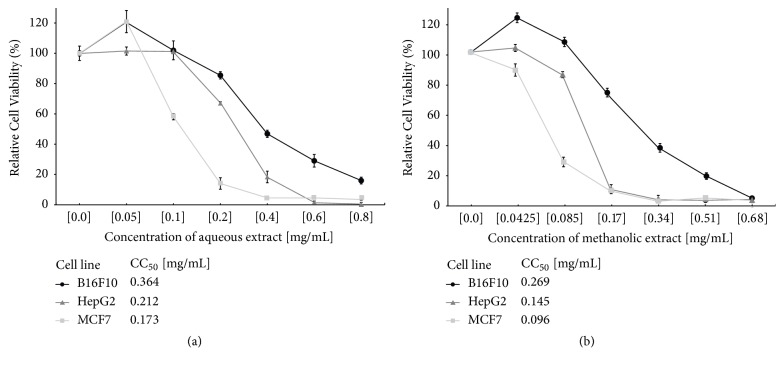
*Effect of aqueous and methanolic extracts of Cuphea aequipetala in cancer cells lines*. The relative cell viability of B16F10 (solid circle), HepG2 (solid triangle), and MCF-7 (solid squared) by MTT read at 48h of exposure to treatments. (a) Effect of the aqueous extract in the cancer cell lines. (b) Effect of the methanolic extract in the cancer cell lines. All treatments were performed in triplicate and the respective standard deviations were included in the graphs.

Acetone-extracts of* C. aequipetala* have shown a cytotoxic effect in human prostate cells (DU-145) [20]; however, the effects of* C. aequipetala* aqueous and methanolic extracts have not been tested. For that reason, we measured the effects of these extracts, starting with the assessment of cell viability on different types of cancer cells lines, which include murine melanoma (B16F10), a human hepatocellular carcinoma (HepG2), and breast cancer (MCF-7). Both aqueous and the methanolic extracts showed cytotoxic activity in a concentration-dependent manner against the cancer cell lines used. The aqueous extract showed an important reduction of cell viability after 48h of exposure; the cytotoxic concentration that induces around 50% of cell death (CC_50_) in B16F10 was 0.364 mg/mL, in HepG2, was 0.212 mg/mL and in MCF-7 was 0.173 mg/mL (Figure 1(a)). The methanolic extract showed even greater reduction of cell viability in all cell lines in comparison to the aqueous extracts, since the CC_50_ of the methanolic extract was in B16F10 of 0.269 mg/mL, in HepG2 of 0.145 mg/mL, and in MCF-7 of 0.096 mg/mL (Figure 1(b)). MCF7 was the most susceptible cell line, obtaining only 14.04% and 9.67% of cell viability at a concentration of 0.2 mg /mL, for the aqueous and methanolic extract, respectively. On the other hand, B16F10 cell line presented a lower sensitivity to the cytotoxic effect of both extracts, obtaining 16.01% of cell viability at the higher concentration of 0.8 mg/mL of the aqueous extract and 8.34% of cell viability using 0.68 mg/mL of the methanolic extract.

### 3.2. Changes in the Morphology of the Cells Treated with* C. aequipetala* Extracts

**Figure 2:**
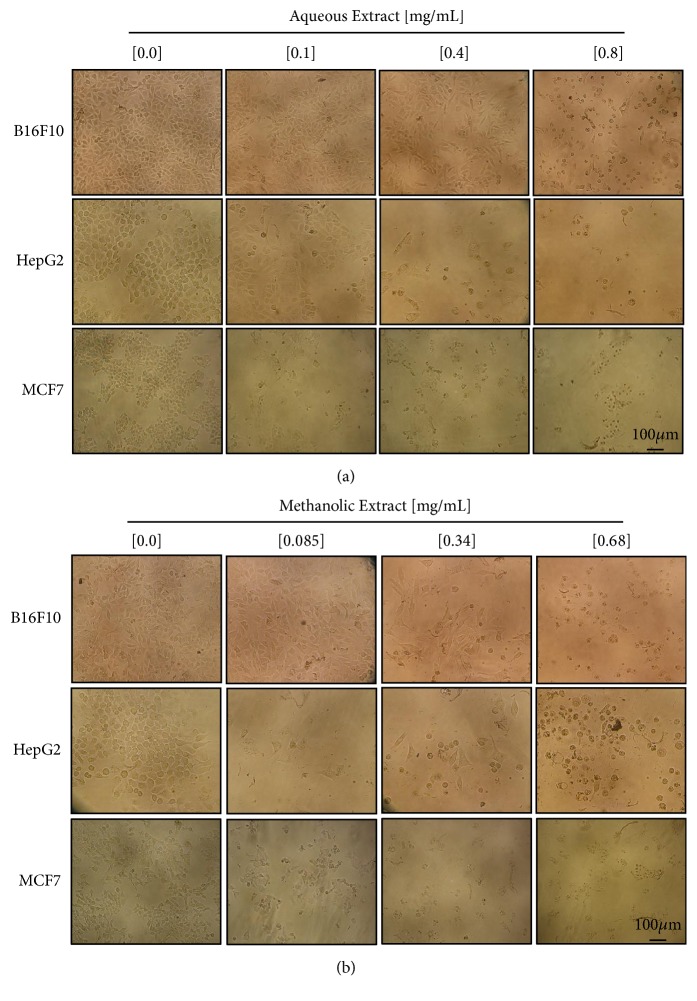
*Effects in the morphology of the cells treated with C. aequipetala extracts*. Effects produced by the (a) aqueous and (b) methanolic extracts of* C. aequipetala*, 48h after incubation on B16F10, HepG2, and MCF7 cells lines at different concentration. The cells were examined under an Olympus inverted microscope using a 20X objective lens. Scale bar: 100*μ*m.

The cytopathic effects caused by increasing concentration of the extracts on B16F10, HepG2, and MCF cell lines were observed with a light microscope after 48h of treatment (Figure 2). In the images is possible to appreciate the morphological changes of each cell line, such as cell volume reduction and cytoplasm shrinkage, and in higher concentrations only the nucleus and cell remnants are observed. Morphological changes are observed from the concentration of 0.1 mg/mL of the aqueous extract (Figure 2(a)) and from 0.085 mg/mL of the methanolic extract (Figure 2(b)). Moreover, karyopyknosis was observed through nuclear Hoechst staining (SF1).

### 3.3. Effect of* C. aequipetala* Extracts in Human PBMCs

**Figure 3:**
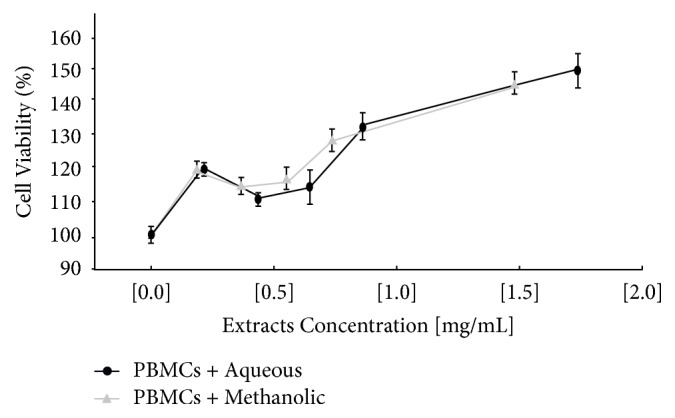
*Effect of aqueous and methanolic extracts of Cuphea aequipetala in human PBMCs.* Analysis of relative cell viability in human PBMCs by MTT analyzed at 48h of exposure. PBMCs were treated with different concentrations of aqueous extract (solid circle) and methanolic extract (solid triangle). The treatments were performed in triplicate and the standard deviation was included in the graph.

Once we determined that the extracts showed cytotoxic effects on cancer cells, we next assessed whether* C. aequipetala* extracts affected the cell viability in noncarcinogenic cells using human PBMCs exposed to different concentrations of both extracts and analyzed after 48 hours of exposure. Cytotoxicity was not found in either extracts, because the MTT assay shows no difference between the control without treatment and the different concentrations used. Conversely, both extracts increased cell viability: the methanolic extract showed an increase of around 50% in viability at a concentration of 1.36 mg/mL, whereas a concentration of 1.6 mg/mL of the aqueous extract was necessary to increase the viability of PBMCs by 50% (Figure 3).

### 3.4. Long-Term Growth Inhibition of B16F10 Cells by Methanolic Extract

**Figure 4:**
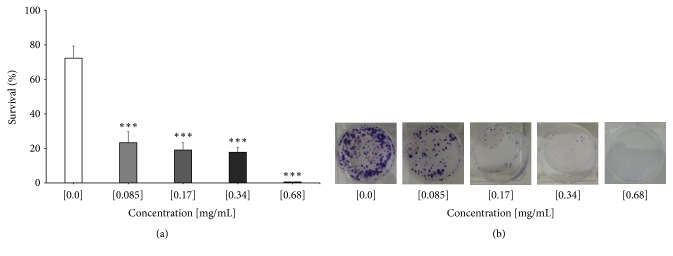
*Analysis of the clonogenic assay of the methanolic extract of C. aequipetala in B16F10 cells.* (a) Analysis of the count of number of clones exposed to the various concentrations of the methanolic extract. The treatments were performed in triplicate and the standard deviation was included. The results shown are representative of triplicates of at least three independent experiments. (b) Representative image of the wells analyzed after treatment with methanolic extract.* p *< 0.05 (*∗*),* p* < 0.01 (*∗∗*), and* p* < 0.001 (*∗∗∗*).

Subsequent analyses of cell death and cytotoxicity were only carried out on B16F10 cells, because of being the cell line used in the murine model and being only analyzed in the methanolic extract that showed a better cytotoxic effect* in vitro*. Continuing with the analysis of the cytotoxic effect of the methanolic extract of* C. aequipetala*, a clonogenic assay was designed. In this assay we are able to show the irreversible damage caused to cells which were exposed to the methanolic treatment for only 24 h and later cultivated during 8 days in culture medium under normal conditions and without treatment. The different concentrations used showed a significant decrease in the number of clones (0.085 mg/mL, 23% (±6.36), 0.17 mg/mL, 19% (±4.24), and 0.34 mg/mL, 17% (±2.83)). The concentration of 0.68 mg/mL showed the highest cytotoxic effect, because no colonies were found at this concentration (Figure 4(a)).  Figure 4(b) shows a representative sample of the reduction in the number and size of the clones surviving in relation to the increase in the concentration of methanolic extract.

### 3.5. Effect of* Cuphea aequipetala* Methanolic Extract on Cell Cycle in B16F10 Cells

**Figure 5:**
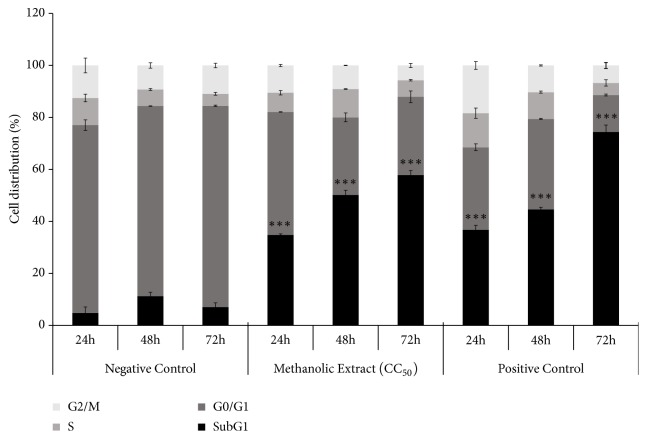
*Effect of Cuphea aequipetala methanolic extract on cell cycle in B16F10 cells*. 1×10^6^ cells were seeded in a 6-well plate and after 24h, cells were treated with the CC_50_ dose of extract (0.269 mg/mL) and etoposide (150*μ*) as the positive control, incubated for 24, 48, and 72 h, and stained with propidium iodide (PI) and samples were acquired by flow cytometer. The results shown are representative of triplicates of at least three independent experiments.* p *< 0.05 (*∗*),* p* < 0.01 (*∗∗*), and* p* < 0.001 (*∗∗∗*).

In an attempt to understand how the* C. aequipetala* extract inhibits B16F10 cell proliferation, the distribution phases of the cell cycle following treatment were analyzed by flow cytometer using Propidium Iodide (PI) staining. After 24, 48, and 72h of incubation, cell cycle analysis revealed that DNA content distribution is altered in the B16F10 cells. The results indicated that the Sub G1 phase was increased by around 30% and 50% when the cells are subjected to longer treatments, suggesting death by apoptosis (Figure 5). The bar graph shows the percentages for G1, S, and G2/M cells. Results are representative of three independent experiments.

### 3.6. Induction of Apoptosis by* Cuphea aequipetala* Extract in B16F10 Cells

**Figure 6:**
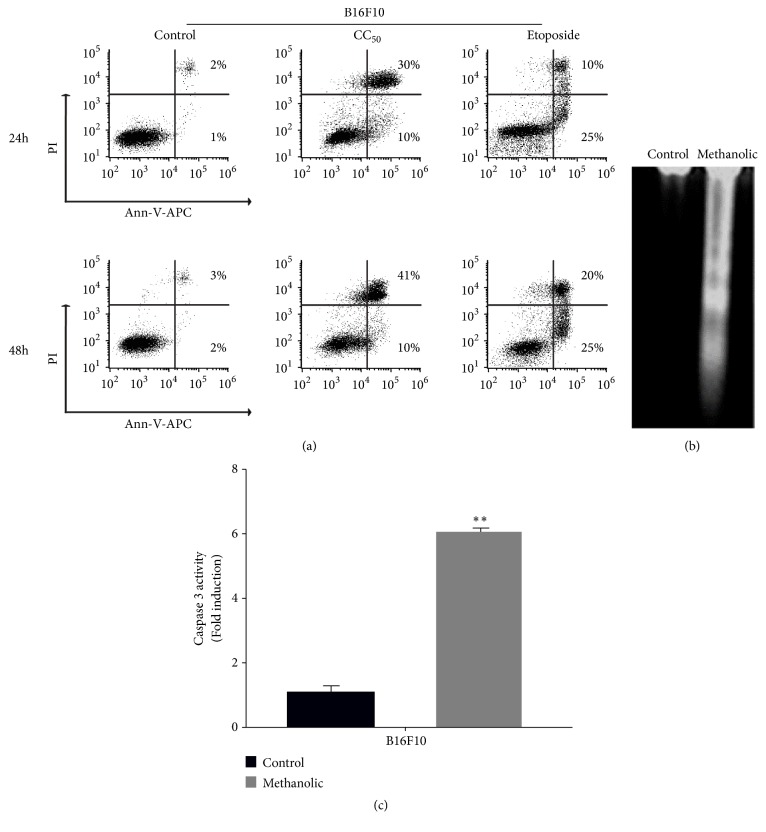
*Induction of apoptosis by Cuphea aequipetala extract in B16F10 cells*. (a) Representative plots of B16F10 cells without treatment (Control) and exposed to methanolic extract (CC50 [0.269 mg/mL]) and etoposide (150*μ*M); in the figure the quadrant distribution (double negative in lower left quadrant, Annexin-V-APC^+^ in lower right quadrant, double positive in upper right as and upper left quadrant as PI^+^) is observed. (b) DNA fractionation assay. Lane 1 corresponds to B10F10 cells without treatment, and lane 2 cells treated with methanolic extract. (c) Caspase-3 activity test. Included cells without treatment (Control) and cells treated with methanolic extract. Bar graphs represent the mean (±SD) of triplicates of at least three independent experiments.* p *< 0.05 (*∗*),* p* < 0.01 (*∗∗*), and* p* < 0.001 (*∗∗∗*).

Previous results pointed that the methanolic extract of* C. aequipetala* induces cell death by apoptosis, for that reason, the next step was to measure the number of Annexin-V-APC/PI positive cells by flow cytometry. The B16F10 cell was treated with 0.135 mg/mL of methanolic extract for 24h obtaining a 50.7 % (±4.94) and for 48h resulting in a 30.95% (±0.91) of Annexin-V-APC/PI cells. The etoposide (150*μ*M, positive control) presented values of 17.80% (±2.12) and 9.43% (±0.55) at 24h and 48h, respectively. Both treatments, methanol extract and etoposide, show higher values (p> 0.05) compared to the control without treatment that presented values of 5.55% (±0.49) and 2.7% (±0.55) (Figure 6(a)). Also, the methanolic extract increased DNA fragmentation, a typical feature of apoptotic cells.  Figure 6(b) shows in lane 1 the negative control (untreated cells), and the DNA fragmentation depicted in lane 2. Moreover, the caspase-3 enzyme activity showed a 6-fold of increase in the treated cells compared with the negative control (Figure 6(c)). The data strongly suggests that the methanolic extract of* C. aequipetala* leads to apoptosis.

### 3.7. The Antitumoral Activity of* C. aequipetala* Extracts* In Vivo* Assay

**Figure 7:**
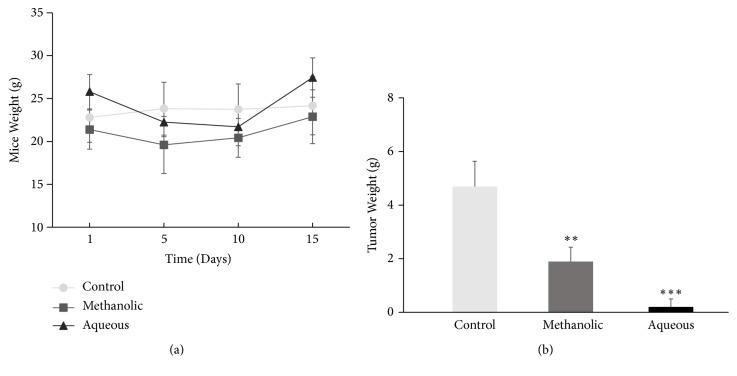
*Effect of Cuphea aequipetala extract in C57BL/6 mice*. A number of 5x10^5^ B16F10 melanoma cells were injected subcutaneously in the dorsal flank. (a) The mice were weighted during the experiment. (b) The weight of tumors collected after 14 days of treatment. The n per each group was 6 mice. Mann-Whitney test was performed.* p *< 0.05 (*∗*),* p* < 0.01 (*∗∗*), and* p* < 0.001 (*∗∗∗*).

To determine the effect of* C. aequipetala* extracts in* in vivo* systems, mice groups were treated with aqueous and methanolic extracts of* C. aequipetala* by adding them in drinking water for 14 days. Neither extracts affect the weight of the mice, since no significant differences in weights were found (Figure 7(a)); however, the treatments showed a reduction in the tumor mass of almost 80% with the aqueous extract, and around 31% with the methanolic extract in comparison to the untreated mice group (Figure 7(b)), which suggest that the aqueous extract of* C. aequipetala* is more effective in a murine melanoma model.

## 4. Discussion

Increased in the incidence of malignant melanoma in susceptible population and the disadvantages presented by anticancer drugs indicate the need to seek new therapeutic alternatives. Herbal extracts are promising candidates: they are safe and effective because of their biological activity and their low toxicity. About 74.8% of all available chemotherapeutic agents are derived from natural products [22]. Plant-derived compounds have active compounds that affect proliferation [23], apoptosis [24], and angiogenesis [25, 26] and can be used against cancer safely, efficiently, and with minimal side effects [27].

The mixture of compounds present in plants can have additive, synergistic, or antagonistic effects that cannot be matched by the isolated compounds, and in some cases, they have a protective effect against the side effects of radio and chemotherapy [28, 29]. In the cosmetic industry, herbal products have been used to inhibit the synthesis of melanin, which shows that it can be an effective natural agent for skin whitening [30, 31]. Plant extracts have shown a cytotoxic effect against human malignant melanoma cells [32, 33], in particular, ethanol extract of* Gardeniae fructus* shows antimetastatic and antiangiogenic activities* in vitro* and* in vivo* in melanoma models [32].

In this work, the cytotoxic effect of aqueous and methanolic extracts of* C. aequipetala* was analyzed in several cancer cell lines to observe its cytotoxic activity, and later the analysis of apoptosis and cell cycle was only carried in the B16F10 melanoma line, with the purpose of subsequently using the* in vivo* model in C57BL/6 mice. In our results, both extracts induce cell death in B16F10, HepG2, and MCF7 cell lines. Previous reports showed the cytotoxic activity of acetone-water extract of* C. aequipetala* in Hep-2 (human laryngeal cancer), HCT-15 (human colon adenocarcinoma), and DU-145 (human prostate cancer) [20]. Also, Vega-Ávila et al. reported that, in a methanolic fraction obtained by them had effect on the HCT-15 and Hep-2 lines, however, our methanolic extract was cytotoxic in all cell lines. Moreover, our methanolic extract showed a greater cytotoxic effect at a lower concentration compared to the aqueous extract* in vitro*. On the other hand, both extracts showed a proliferative effect in normal cells (PBMC). This proliferative effect may be due to the fact that the extracts may contain sugars and antioxidants, which help in the proliferation of normal cells [34]. Phytochemical characterization of the* C. aequipetala* extract showed phenols, terpenes, steroids, and saponins and some components presented antioxidant and antilipase activities [34].

The treated cells also presented morphological changes that indicate a possible death by apoptosis, because some characteristics of this type of cell death were present in the treated cells, like cytoplasmic contraction, nuclear fragmentation (karyorrhexis), and formation of discrete corpses that initially retain the integrity of the plasma membrane (apoptotic bodies) [35]. Apoptosis induction by anticancer compounds is considered an important goal [36, 37]. Studies have shown that extracts exert their cytotoxic properties through apoptosis [24, 38, 39]. Our results showed that the methanolic extract induces the cell death of B16F10 cells via apoptosis, because we observed an increase in Annexin-V and PI positive cells, DNA fragmentation, and activation of caspase 3 in cells treated with this extract. Moreover, the effect of the methanolic extract in the cell cycle was analyzed and a higher accumulation in sub G1 phase was observed, indicating cellular arrest. Similar results were reported using* Rubus fairholmianus* and* Ginko biloba, *which inhibit melanoma cell growth by the caspase-dependent apoptotic pathway [39, 40].

The* in vivo* results are promising because just drinking the extracts as regular drinking water, both extracts caused a reduction in tumor mass in a melanoma murine model, proving the antiproliferative and antitumor effects of* C. aequipetala* extracts. It should be mentioned that there was no statistically significant difference when measuring the volume consumed by the mice of both groups; however, due to the mice behavior, it was easy to assume that the methanol extract was not to their liking compared to the aqueous extract, which may be a factor by which the aqueous extract worked better than methanol in the in vivo model. There are several* in vivo* studies of melanoma treated with plant extracts with satisfactory results [41, 42]. Our preliminary studies suggest that extracts of* C. aequipetala* are nontoxic in mice at least 14 days after treatment, because they did not cause significant changes in weight and other clinical manifestations such as diarrhea by treatment intake; also, mice showed no alterations in blood count (data not shown). Overall, our results highlight the potential therapeutic use of* C. aequipetala* extracts targeting cancer. However, the compounds, which are inducing the cell death remains unclear, as does whether this type of treatment, could be used therapeutically. Therefore, we believe that* C. aequipetala* and their products deserve further investigation.

## 5. Conclusions

Based on the results obtained from this study, we have demonstrated that extracts of* C. aequipetala *reduced proliferation in B16F10 cell line* in vitro* and increased Sub G1 phase and induced cell death, apparently by apoptosis. The oral administration of aqueous and methanolic extract of* C. aequipetala* reduced tumor weight in a murine melanoma model. Preliminarily, the extracts of* C. aequipetala* can be stated as a potential therapeutic option in cancer.

## Data Availability

The data and materials supporting the conclusions of this article are included within the article.

## Supplementary Materials

Supplementary Materials Supplementary Figure 1.pdf: nuclear effect of methanolic extract on B16F10. (A) Nuclear staining with Hoechst 33258, at upper left, merge of visible light with Hoechst staining of control cells without treatment, upper right Hoechst staining, and bottom left merge of visible light with Hoechst staining of treated cells with methanolic extract (CC50). (B) Graph of the percentage of karyopyknosis in B16F10 cells without treatment and treated with the methanolic extract of (C) aequipetala at a concentration of 269 *μ*g/mL (CC50), p = 0.001. Bar graphs represent the mean (±SD) of triplicates of at least three independent experiments.
