# Supplementary material for: Inhibitory Effect of Cuphea aequipetala Extracts on Murine B16F10 Melanoma In Vitro and In Vivo
Source: Biomed Res Int. 2019 May 29;2019:8560527. doi: 10.1155/2019/8560527 (PMC6560323; doi:10.1155/2019/8560527)
Supplement: Supplementary Materials — Supplementary Figure 1.pdf: nuclear effect of methanolic extract on B16F10. (A) Nuclear staining with Hoechst 33258, at upper left, merge of visible light with Hoechst staining of control cells without treatment, upper right Hoechst staining, and bottom left merge of visible light with Hoechst staining of treated cells with methanolic extract (CC50). (B) Graph of the percentage of karyopyknosis in B16F10 cells without treatment and treated with the methanolic extract of (C) aequipetala at a concentration of 269 μg/mL (CC50), p = 0.001. Bar graphs represent the mean (±SD) of triplicates of at least three independent experiments. [file 8560527.f1.zip › mat.8560527.v2.pdf]

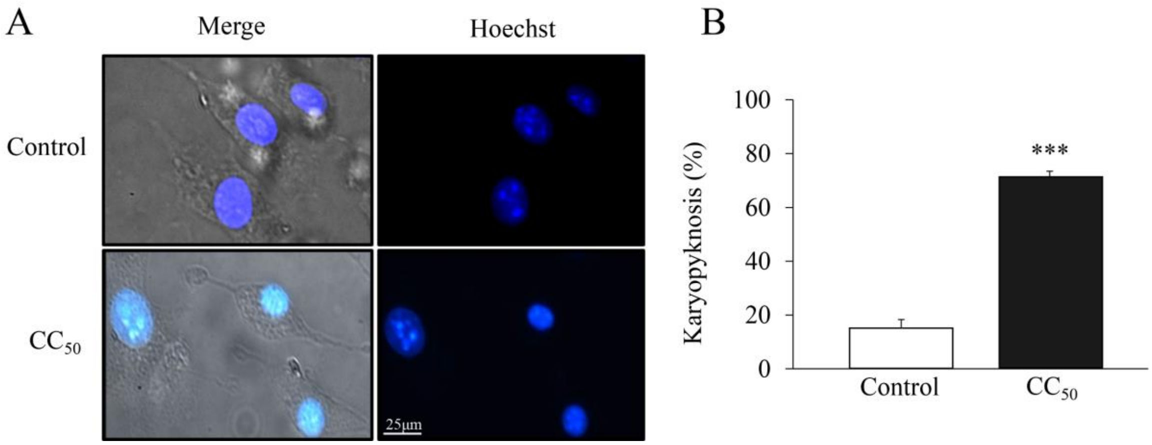

Supplementary figure 1. Nuclear effect of methanolic extract on B16F10. A. Nuclear staining with Hoechst 33258, at upper left, merge of visible light with Hoechst staining of control cells without treatment, upper right Hoechst staining, bottom left merge of visible light with Hoechst staining of treated cells with methanolic extract (CC_{50}). B. Graph of the percentage of karyopyknosis in B16F10 cells without treatment and treated with the methanolic extract of *C. aequipetala* at a concentration of 269 µg/mL (CC_{50}), $p = 0.001$. Bar graphs represent the mean (±SD) of triplicates of at least three independent experiments.
